# 3D MRI Reconstruction Based on 2D Generative Adversarial Network Super-Resolution

**DOI:** 10.3390/s21092978

**Published:** 2021-04-23

**Authors:** Hongtao Zhang, Yuki Shinomiya, Shinichi Yoshida

**Affiliations:** 1 Graduate School of Engineering, Kochi University of Technology, Kami, Kochi 782-8502, Japan; 2 School of Information, Kochi University of Technology, Kami, Kochi 782-8502, Japan; shinomiya.yuki@kochi-tech.ac.jp (Y.S.); yoshida.shinichi@kochi-tech.ac.jp (S.Y.)

**Keywords:** magnetic resonance imaging, deep learning, super-resolution, three-dimensional reconstruction, RFB-ESRGAN, nESRGAN

## Abstract

The diagnosis of brain pathologies usually involves imaging to analyze the condition of the brain. Magnetic resonance imaging (MRI) technology is widely used in brain disorder diagnosis. The image quality of MRI depends on the magnetostatic field strength and scanning time. Scanners with lower field strengths have the disadvantages of a low resolution and high imaging cost, and scanning takes a long time. The traditional super-resolution reconstruction method based on MRI generally states an optimization problem in terms of prior information. It solves the problem using an iterative approach with a large time cost. Many methods based on deep learning have emerged to replace traditional methods. MRI super-resolution technology based on deep learning can effectively improve MRI resolution through a three-dimensional convolutional neural network; however, the training costs are relatively high. In this paper, we propose the use of two-dimensional super-resolution technology for the super-resolution reconstruction of MRI images. In the first reconstruction, we choose a scale factor of 2 and simulate half the volume of MRI slices as input. We utilize a receiving field block enhanced super-resolution generative adversarial network (RFB-ESRGAN), which is superior to other super-resolution technologies in terms of texture and frequency information. We then rebuild the super-resolution reconstructed slices in the MRI. In the second reconstruction, the image after the first reconstruction is composed of only half of the slices, and there are still missing values. In our previous work, we adopted the traditional interpolation method, and there was still a gap in the visual effect of the reconstructed images. Therefore, we propose a noise-based super-resolution network (nESRGAN). The noise addition to the network can provide additional texture restoration possibilities. We use nESRGAN to further restore MRI resolution and high-frequency information. Finally, we achieve the 3D reconstruction of brain MRI images through two super-resolution reconstructions. Our proposed method is superior to 3D super-resolution technology based on deep learning in terms of perception range and image quality evaluation standards.

## 1. Introduction

The diagnosis of brain lesions is an active field of research. Magnetic resonance imaging (MRI) is one of the most important diagnostic imaging methods and has been widely used in diagnosis and image-guided treatment, especially for brain imaging diagnosis. Through brain MRI, brain abnormalities and lesions can be observed. MRI scanners with higher magnetic field strength provide higher signal-to-noise ratio (SNR) [1,2,3,4]. At present, the 1.5 T MRI scanner is a commonly used instrument in hospitals. Compared with a 3 T MRI scanner, the time cost of image acquisition using a 1.5 T MRI scanner is higher because its SNR is lower. Due to imaging technology limitations, such as high SNR and long scanning time, image super-resolution (SR) is favored by medical experts [5].

Early research on super-resolution MRI images used super-resolution reconstruction (SRR) to improve the image resolution. SRR combines a series of low-resolution MRI images into a high-resolution image [5,6,7]. This method requires large time and equipment costs, and subsequent research has shown that adding more low-resolution scans does not necessarily improve the resolution [8,9]. Subsequently, with the introduction of single-image super-resolution (SISR) [10,11], MRI super-resolution reconstruction only requires low-resolution scans corresponding to high-resolution output. Initially, SISR used a form of regularization conditions and then used prior knowledge to enhance the reconstruction ability of linear models [11]. However, this type of method is computationally complex and requires many computing resources [5,7,12].

Based on the convolutional neural network (CNN) model, the super-resolution CNN (SRCNN) [13] was introduced into SISR. MRI super-resolution research attempted to combine convolutional neural networks for super-resolution reconstruction [5,7,8,9]. With the introduction of residual networks [14,15,16,17,18,19], super-resolution technology based on deep learning began to develop into deeper networks [14,15,16]. However, the reconstructed images are usually too smooth and lack visual authenticity, depending on the mean square error (MSE) loss function. With the introduction of generative adversarial networks (GANs) [20], the blurring image problem is improved, and SISR has been developed to reduce perceived loss [19]. MRI super-resolution reconstruction is focused on GANs to restore more textural details and high-frequency information rather than overall clarity.

**Figure 1:**
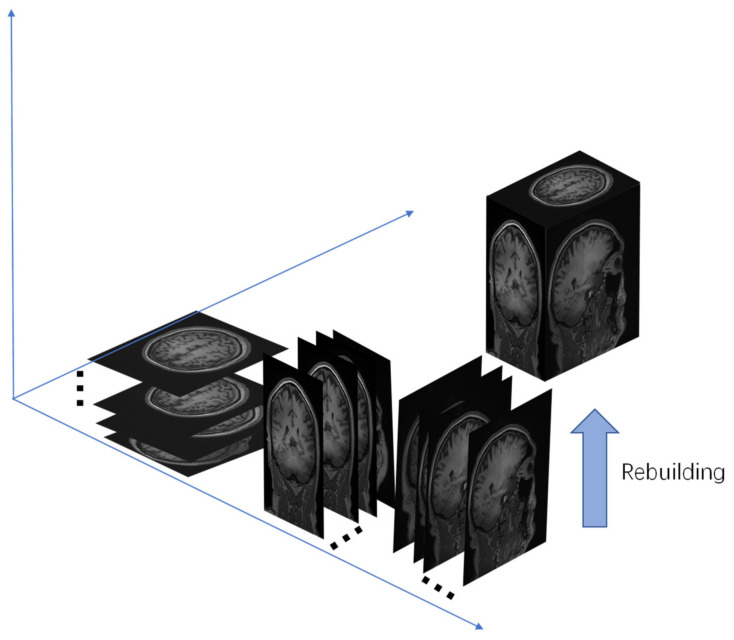
Rebuilding MRI from three plane slices. Number of slices is half that of MRI. Number of slices in three directions is 128 (256 × 150 pixels), 128 (256 × 150 pixels), and 75 (256 × 256 pixels).

**Figure 2:**
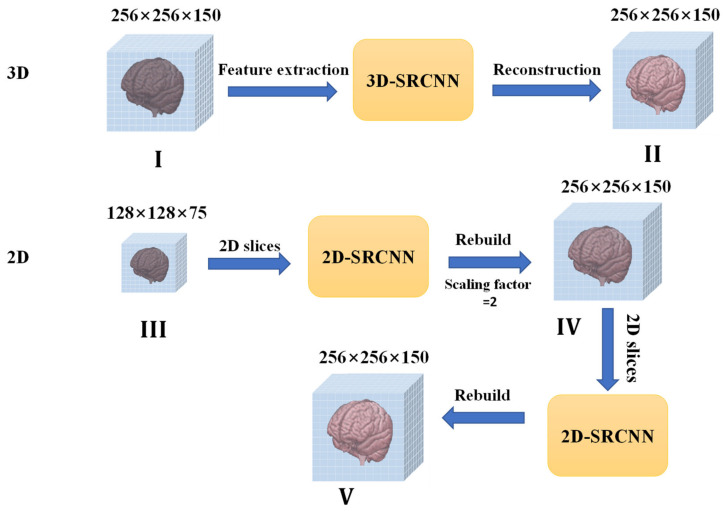
Main processes of proposed method. I and II: MRI super-resolution reconstruction for three-dimensional convolutional neural network (3D-CNN). Our method includes III, IV, and V. Depending on scaling factor, input shape is half of MRI (128 × 128 × 75), which consists of 128 (128 × 75), 128 (128 × 75), and 75 (128 × 128) slices. In process IV, through two-dimensional super-resolution CNN (2D-SRCNN), MRI can be recovered with more detail and rebuilding can be finished.

We consider that MRI is stored in a three-dimensional matrix. MRI super-resolution reconstruction is usually combined with a three-dimensional convolutional neural network (3D-CNN), which can directly extract 3D image features to reconstruct the entire MRI image. However, it can be computationally complex and cause memory allocation problems. Therefore, we propose using 2D CNNs to replace 3D CNNs in order to reduce memory and time costs. Combined with a scale factor of 2, the entire MRI data reconstruction should be a half-volume MRI (Figure 1, 2D in Figure 2), so we preprocessed half number of the MRI slices as a dataset in the slice processing. We rebuilt the reconstructed slices in the three planes into an MRI image to complete the first reconstruction step (Figure 1). Because MRI involves reconstructing half the number of slices in three planes, there are missing values in the reconstructed MRI. Therefore, we propose the second super-resolution reconstruction work for detail restoration. After two super-resolution reconstructions, we realized three-dimensional MRI reconstruction in the two-dimensional super-resolution convolutional neural network.

The main contributions of this paper include the following:

(1) A new noise-based enhanced super-resolution generative adversarial network (nESRGAN) with the addition of noise and interpolated sampling is proposed. The noise part of the network can provide specific high-frequency information and details without affecting the overall feature recovery. Simultaneously, interpolation sampling solves artifacts and color changes caused by the checkerboard effect [21].
(2) Our proposed method is better than the super-resolution method based on 3D neural networks in respect of the reconstruction effect. The high-resolution MRI images can assist doctors in obtaining more detailed brain information, which is of particular significance for diagnosing and predicting brain diseases by using a 1.5 T MRI scanner.

The related methods are outlined in Section 2. Section 3 introduces the relevant configuration and parameters of the study. In Section 4, we present the results and analysis. In Section 5, we discuss the experimental data and results, summarize the experiment, and describe the main work planned for the future.

## 2. Main Method of Reconstruction

### 2.1. Main Idea and Processes

In most super-resolution studies of MRI, researchers often use three-dimensional convolutional neural networks for image reconstruction [22,23,24]. However, the three-dimensional convolutional neural networks has excessive parameters and large weight, which results in a considerable memory cost. An MRI image is usually saved in the form of a voxel matrix. According to the matrix’s particularity, MRI rebuilding can be completed with half the number of slices in three directions, but there are many missing values (as shown in Figure 1). In 3D-SRCNN, MRI structure information is generally read to perform reconstruction (as shown in the 3D-SRCNN in Figure 2). Using a two-dimensional CNN to replace a three-dimensional CNN can reduce computational costs. As shown in Figure 2, our study’s scaling factor is 2, and thereby we simulate half of volume MRI as the dataset. Compared with whole MRI data, it only includes a half number of slices in three planes. We adopted the MRI slice operation. After reconstruction from 2D-SRCNN, new MRI data is rebuilt by these reconstructed slices (Figure 1). Since the reconstructed MRI only contains half the number of slices, there have been missing values. For the problem of missing values, we can use the new 2D-SRCNN network for further recovery. In the second step of recovery work, we utilize the same slicing operation in new 2D-SRCNN and obtain a new MRI consisting of newly reconstructed slices. After the first and second steps, we succeeded in replacing the 3D-SRCNN and completed the super-resolution reconstruction of the MRI. In general, we propose two neural networks to perform reconstruction work. The first reconstruction work is based on the structural particularity of the MRI voxel matrix. Half the number of slices (half the volume) are selected for super-resolution reconstruction and MRI rebuilding. The second reconstruction work uses super-resolution technology to further repair the missing values of the reconstructed MRI to complete the rebuilding.

In deep learning, MRI super-resolution reconstruction technology is based on learning high-resolution image features from a great deal of MRI data [25,26]. In recent years, the use of generated adversarial neural networks (GANs) for super-resolution reconstruction has become the mainstream [25]. With GANs, restored images have more detailed features. Among many super-resolution adversarial neural networks, ESRGAN with a receptive field module is superior to other methods in restoring high-frequency details and maintaining content consistency. Receiving field block (RFB)-ESRGAN [19] can obtain more detailed information for brain MRI (Table 1). Therefore, we adopted RFB-ESRGAN in the first reconstruction work. Taking into account the interactivity of MRI in three planes, each slice in the rebuilt MRI image has missing values due to insufficient pixel information.

**Figure 3:**
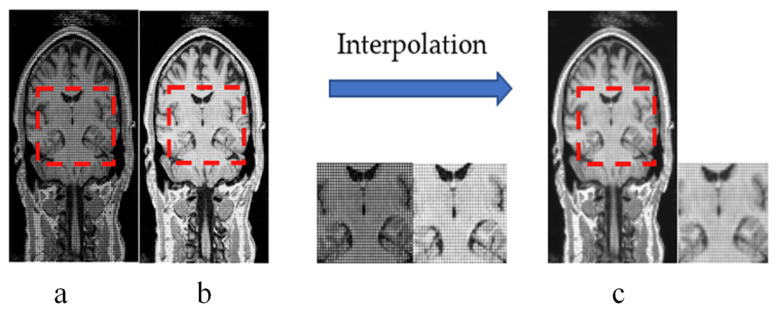
(**a**,**b**) Adjacent slices which are from with different brightness; Slices belong to the newly reconstructed MRI in the first reconstruction. (**c**) New slice after interpolation repair. Noise and grid lines still exist from traditional interpolation. Interpolation only solves the problem of brightness and recovers only a few details.

In the experimental part of our study, the primary process of 3D reconstruction is to perform two super-resolution reconstructions. In the first reconstruction, we use an expansion parameter whose size is 2. Therefore, the entire MRI involved in training should also be half the volume. After performing super-resolution reconstruction on low-resolution two-dimensional slices, rebuilding is performed. The reconstituted MRI image has only a half number of the slices in all three planes. Considering the interactivity, each slice in this new image has missing values. In our previous work, we referred to linear interpolation and used surrounding non-zero pixels in the zero value of each slice and then performed substitution and interpolation in the second reconstruction. However, we found that there were differences in brightness between the reconstituted brain MRI slices. At the same time, traditional interpolation repair can only solve the issue of brightness. There are still noise and missing values in the interpolated slices (as shown in Figure 3), so we propose a noise-based network (nESRGAN) to perform the second super-resolution reconstruction. Inspired by Style-GAN [28], we found that the addition of noise can help in restoring features and supplementing more high-frequency information. Similarly, ESRGAN also causes the checkerboard effect as a result of deconvolution [21]. Therefore, we propose an interpolation sampling recovery block to replace the deconvolution layer so that the reconstructed image no longer has a checkerboard pattern of different colors.

**Figure 4:**
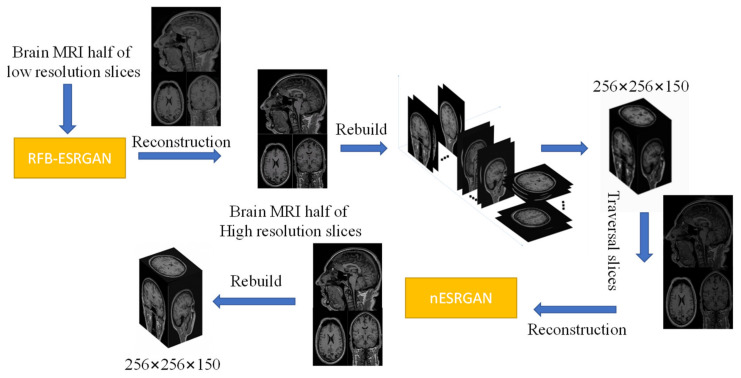
Main processes of experiment. Prepare slices on three planes (with an interval of 1 slice) for super-resolution reconstruction. Obtain high-resolution MRI slices through receiving field block enhanced super-resolution generative adversarial network (RFB-ESRGAN). Since there is half the number of slices, they can still be rebuilt into the MRI. Rebuilt MRI image has many missing values because half the slices are missing; then, super-resolution reconstruction is used to repair the image. Finally, super-resolution reconstruction is completed.

We use RFB-ESRGAN [19] for the first MRI reconstruction. After that, half a number of the MRI slices are reorganized to obtain an image with much noise and many missing values. Then we use nESRGAN to perform the super-resolution reconstruction, and finally rebuild a new high-resolution MRI image. The main processes are shown in Figure 4.

### 2.2. MRI Slice Reconstruction Based on RFB-ESRGAN

**Figure 5:**
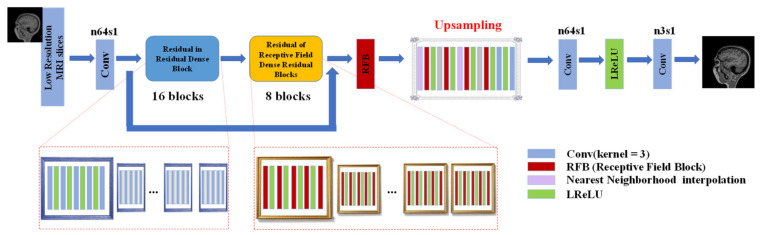
Generator network with k = 3 kernel size, n = (3, 64, 256) feature maps, and s = 1 stride. Network has 2 residual dense blocks, RRDB has 16 blocks, and RRFDB has 8 blocks in the whole network. LReLU is activation function.

To obtain more MRI details and reduce network complexity, we propose RFB-ESRGAN for the first MRI super-resolution reconstruction. The main structure of the network is shown in Figure 5.

RFB-ESRGAN alternately uses the upsampling operations of nearest neighborhood interpolation (NNI) and sub-pixel convolution (SPC) to achieve a good blend of spatial and depth information that will not lose detail performance due to over-resolution. Alternating different upsampling methods reduces the computational complexity. To a certain extent, the super-resolution of multi-scale MRI images can be achieved.

**Figure 6:**
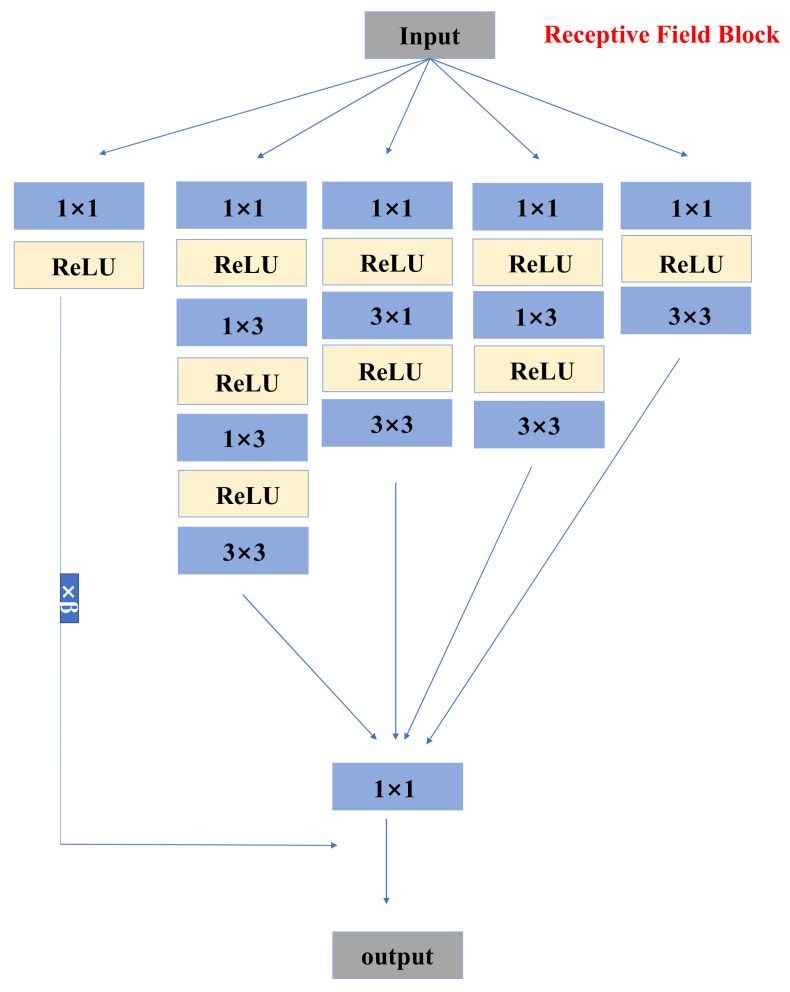
Structure of receptive field block (RFB). Block includes numerous small size kernels. RFB can provide detailed information. ReLU is activation function in network.

In terms of generators, the network mainly includes the residuals in the dense residual blocks and the receptive field blocks. The residual receptive field block uses small convolution kernels of different sizes for detail restoration, reducing the number of model parameters and computational complexity. The network introduces receptive field blocks (RFBs) [19,29] to super-resolution, which balances the problems of small calculation and large receptive field, and can extract very detailed features, thereby obtaining more detailed textures of the MRI image. The network structure of the residual receptive field block is shown in Figure 6.

**Figure 7:**
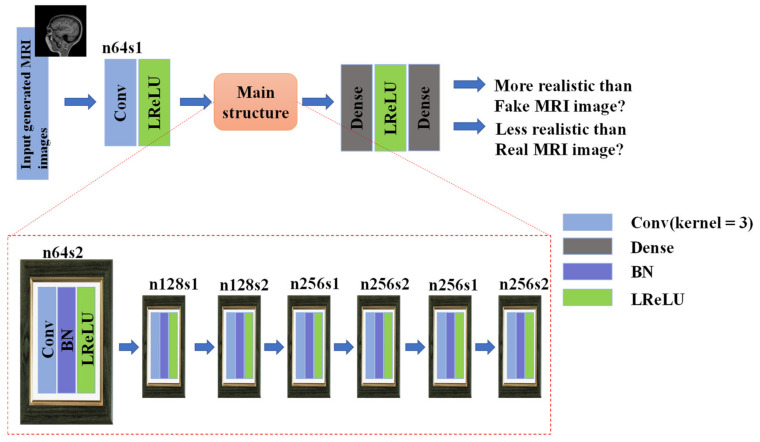
Discriminator network with k = 3 kernel size, n = (64, 128, 256, 512) feature maps, and s = (1,2) stride. In this network, dense is fully connected layer, BN is Batch Normalization and LReLU is activation function.

In terms of the discriminator, the network still uses the idea of Ra-GAN [30] to calculate the more realistic probability of the reconstructed MRI. The main structure is shown in Figure 7.

After setting up the network, we adopted two-stage training. The first stage uses L1 loss for training, then uses the second stage to introduce content loss and adversarial loss to fine-tune the model to avoid instability in the training process. Through the trained RFB-ESRGAN, we reconstructed half of the MRI images in three directions in each MRI. Then we used these MRI images to reconstruct a new MRI (first step in Figure 4).

### 2.3. MRI Slice Reconstruction Based on nESRGAN

**Figure 8:**
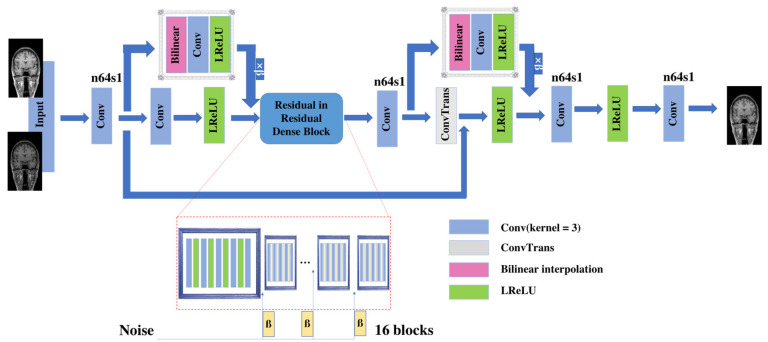
Generator network with k = 3 kernel size, n = (3, 64) feature maps, and s = 1 stride. Network has 16 residuals in residual dense blocks. ConvTrans is deconvolution layer. LReLU is activation function.

To recover detailed information, we used nESRGAN for the second MRI reconstruction. The specific structure of the network is shown in Figure 8.

nESRGAN uses ESRGAN as the leading architecture. Aiming at the missing values in MRI, we added noise to the residuals in the residual dense block to generate certain detailed image information. At the same time, in order to avoid the checkerboard effect, we added interpolation sampling. The sampling block replaces the original deconvolution layer [21] to avoid artifacts. The slice does not need to be degraded, so when the scaling factor exists, we can add the sampling block in the feature extraction link to achieve down-sampling and obtain sufficient feature information. The discriminator network is consistent with RFB-ESRGAN (Figure 7).

In the training of nESRGAN, our dataset is sliced from the reconstructed MRI. The network includes a downsampling module, so there is no need for image degradation processing. We only need to traverse all MRI slices and then reconstruct a new image through the trained nESRGAN (the second step in Figure 4).

### 2.4. Related Loss Function

The network loss used in our methods is consistent with ESRGAN. The generator loss part is composed of perceptual loss, adversarial loss, and pixel loss. The perceptual loss uses the VGG-19 [31] before activation as the extraction feature. The adversarial loss is the value of loss against the Ra-GAN [30] discriminator, and the pixel loss is the *L*1 of the supervised learning enhancement output and the label [18,19,32]. The generator loss (*L_G_*) can be expressed as follows:

(1) $$L_{G}=L_{\mathrm{percep}}\;+\lambda L_{G}^{Ra}\;+\eta l_{1}$$

*L*_percep_ = ∑ ||*VGG(I^SR^)* − *VGG(I^HR^)* ||_1_ (2)

*L*_percep_ is perceptual loss, which includes VGG adversarial loss (*VGG(I^SR^*, *I^HR^*). λ and η are the coefficients to balance different loss terms. $L_{G}^{Ra}$ reflects the probability that real images are relatively more realistic than fake images. *l*_1_ is pixel loss. $L_{G}^{Ra}$ can be expressed as follows:

*I*^SR^ = *G*(*I^LR^*) (3)

(4) $$L_{G}^{Ra}=\sum \;||I^{\mathrm{SR}}-I^{\mathrm{HR}}|{|}_{1}$$

The loss of pixels is as follows:

*l*_1_ = −*E* [log (*1-*∆*_Real_*)] − *E* [log(∆*_Fake_*)] (5)

∆*_Real_* = sigmoid(*D*(*I^HR^*) − *E* [*D(I^SR^*)]) (6)

∆*_Fake_* = sigmoid(*D(I^SR^*) − *E* [*D(I^HR^*)]) (7)

In terms of the discriminator, the idea of Ra-GAN is adopted. It mainly determines whether a picture is more real than another picture; that is, the real image is relatively more realistic than the fake image. The loss function is expressed as follows:

(8) $$L_{D}^{RaSGAN}=-E_{x_{r}\sim P}\left[\log\left(\underline{D}\;\left(x_{r}\right)\right)\right]-E_{x_{f}\sim Q}\left[\log\left(1-\underline{D}\left(x_{f}\right)\right)\right]$$

$$x=\mathrm{sigmoid}\left(C\left(x\right)-E_{x_{f}\sim Q}C\left(x_{f}\right)\right)\mathrm{if}\;x\;\mathrm{is}\;\mathrm{real}$$

(9) $$x=\mathrm{sigmoid}\left(C\left(x\right)-E_{x_{r}\sim P}C\left(x_{r}\right)\right)\mathrm{if}\;x\;\mathrm{is}\;\mathrm{fake}\;$$

P is the distribution of real data. Q is the distribution of fake data. $x_{f}$ is the image generated by the generator, and $x_{r}$ is the original image. $C\left(x\right)$ and $\underline{D}\left(x\right)$ are, respectively, defined as the output of the non-transform discriminator and the standard discriminator.

### 2.5. Image Quality Evaluation Indicators

Traditional MRI super-resolution reconstruction techniques generally follow the MSE-based peak signal-to-noise ratio (PSNR) [33] as the reconstructed image quality index. However, PSNR is based on error-sensitive image quality evaluation, in which the visual characteristics of the human eye are not considered. As a result, the evaluation results are often inconsistent with people’s subjective feelings. As a result, images are too smooth and lack high-frequency information. For this reason, we added structural similarity (SSIM) [33] and learned perceptual image patch similarity (LPIPS) [34] as the evaluation criteria for reconstructed images. The higher the values of PSNR and SSIM, the lower the distortion of the picture. LPIPS [34] is used to evaluate the distance between image patches; the lower the value of LPIPS, the more similar the images.

PSNR and SSIM [33] can be expressed as follows:

(10) $$\mathrm{PSNR}=10\;\times \;\log_{10}\left(\frac{{\mathrm{MAX}}_{I}^{2}}{MSE}\right)$$

(11) $$\mathrm{SSIM}(x,y)=\frac{\left(2\mu_{x}\mu_{y}+c_{1}\right)\left(2\sigma_{xy}+c_{2}\right)}{\left(\mu_{x}^{2}+\mu_{y}^{2}+c_{1}\right)\left(\sigma_{x}^{2}+\sigma_{y}^{2}+c_{2}\right)}$$

where *μ_x_* and *μ_y_* represent the mean values of images X and Y, *σ_x_* and *σ_y_* represent the variances of images X and Y, and *σ_xy_* represents the covariances of images X and Y.

## 3. Comparisons and Configuration

### 3.1. Experimental Configuration

#### 3.1.1. Dataset

All MRI data comes from the IXI (Information eXtraction from Images) dataset, which includes structural MRI of 581 healthy adults. In this study, we use T1-weighted structural images. The dataset is freely available from the following website: http://brain-development.org/ixi-dataset/ (accessed on 20 August 2020).

This study uses T1 brain MRI data, with a primary size of 256 × 256 × 150 pixels, and the main data format is NIfTI. A total of 581 pieces of T1-weighted brain data were used; We divided the dataset according to the ratio of 7:3. 431 pieces of data as the training set, and the remaining 150 as the test set. In preparing the dataset at the beginning of the experiment, we performed degradation processing of low-resolution images through the bicubic difference. The size of slices on the three planes was half the original size. We divided each slice into multiple image patches. The sizes of the paired LR and HR images were 16 × 16 and 32 × 32, respectively.

In the first reconstruction of this research, we adopted sampling slices with an interval of 1 slice and obtained half the slices on three planes. After that, we redivided the reorganized MRI images into the training set and testing set. After reorganizing 150 images, 120 were used as the training set and 30 as the test set. In the second reconstruction work, we directly used all the slices on the three planes as training and completed the image patching work.

#### 3.1.2. Experimental Environment

Throughout the whole experiment, we built the model under the Pytorch framework and used GPU for network training. The main configuration was a Tesla V100-SXM2 (32 GB) DGX system.

#### 3.1.3. Experimental Configuration

**Figure 9:**
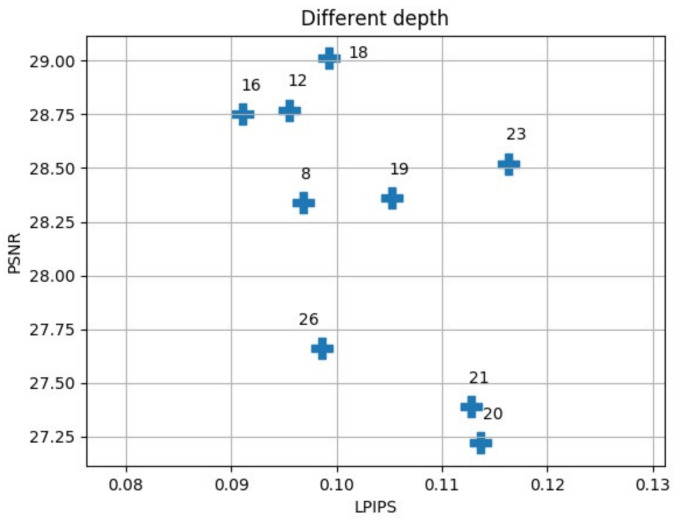
Different network depths for super-resolution in this study; 16 blocks performs better than other depths by PSNR and LPIPS. With small network depth, 16 and 12 blocks also perform best. With large network depth, 23 perform best.

**Figure 10:**
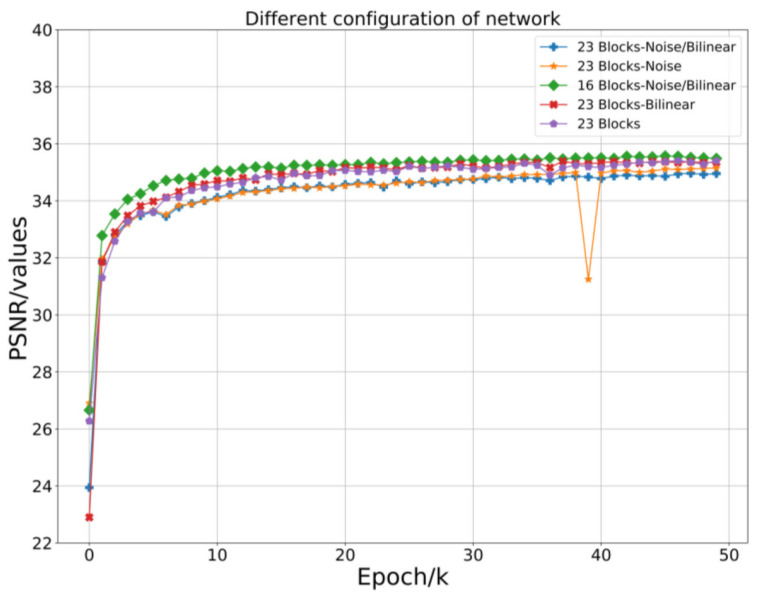
Comparison of different depths and structural networks. There is only a noise part compared to the original network, and PSNR is lower. At the same time, the interpolation sampling part is also relatively lower. In both cases, the greater the network depth, the lower the PSNR. The 16-block network with the noise part and interpolation sampling part performs best.

When building a residual network, we considered reducing the network parameters while maximizing the network’s efficiency. We compared the situation of the network at different depths (Figure 9 and Figure 10).

The deeper the network we chose, the better the performance. However, there were also network parameters and calculations that increased. For this reason, we needed to use as little depth as possible while maintaining better efficiency. Therefore, we chose 16 residuals with relatively good performance as the network depth. In the network construction of RFB-ESRGAN, we used 16 residuals in the residual deep network blocks and 8 perceptual field blocks [19,29]. In addition, in nESRGAN, we added an interpolation sampling method in the feature extraction part and the upsampling of the network. At the same time, we added noise to the network to recover detailed information [35]. In Figure 9, 23 is the best performer in the deeper network. Therefore, we compared networks with smaller and larger depths, and we selected and compared the residual network’s performance with 16 and 23 (Table 2). The depth of 16 is superior to 23 in all evaluations.

In the experiment, the batch sizes of the two networks were both suitable for 16 blocks. In the noise part of nESRGAN, the network uses Gaussian noise [28]. We used linear interpolation for feature extraction and upsampling (Figure 6). This setting can avoid the checkerboard effect, while providing more detailed functions.

### 3.2. Comparison

#### 3.2.1. RFB-ESRGAN

**Figure 11:**
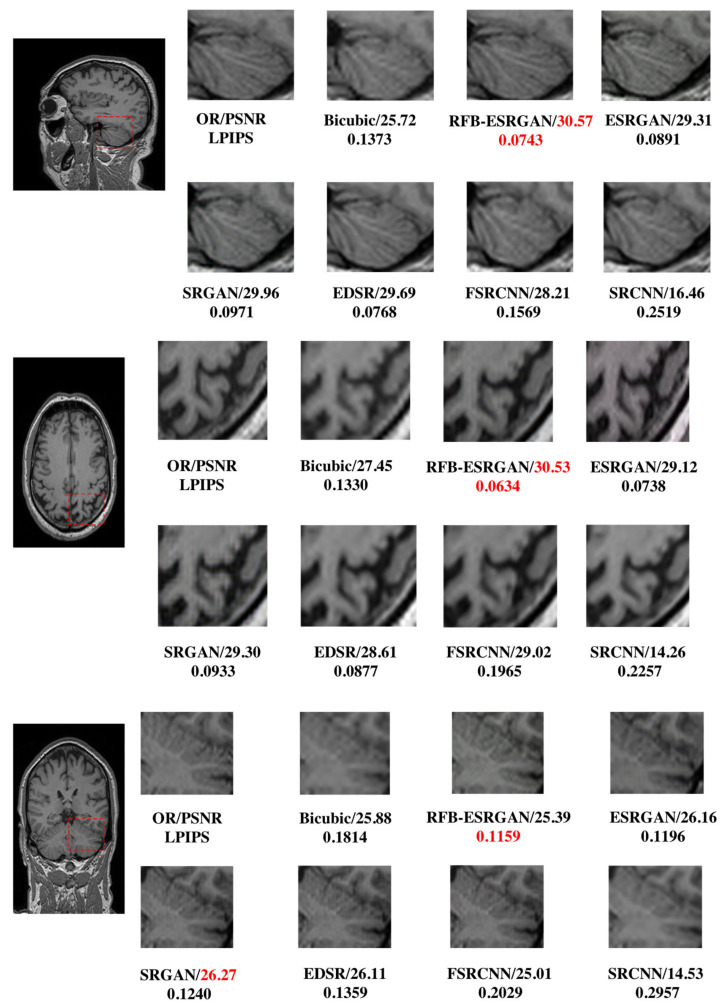
Comparison of SRCNN [36], FSRCNN [27], EDSR [15], SRGAN [17], ESRGAN [18], RFB-ESRGAN [19] in the first reconstruction.

We chose RFB-ESRGAN in order to verify its advantages of high-detail information, and compared it with the traditional super-resolution methods in deep learning (Table 1). We found that RFB-ESRGAN performed best in image evaluation indicators and visual quality overall (Figure 11).

#### 3.2.2. nESRGAN

In the second reconstruction, considering that we hoped for better performance in the image quality evaluation, we tried to compare the situation after the second reconstruction under different configurations. We separately set the noise and interpolation sampling part, no noise and interpolation sampling part, only the noise part, and only the interpolation sampling part. We considered whether the difference in network depth would affect image reconstruction quality; we also set two depth networks: 16 and 23. We compared them (Table 2) and found that the noise and interpolation sampling part with a depth of 16 performed best. The PSNR comparison chart is shown in Figure 7. For this reason, we selected nESRGAN with a depth of 16 as the second reconstruction network.

## 4. Results

### 4.1. First Super-Resolution Reconstruction

We tested the slices of three planes in the first super-resolution process, and RFB-ESRGAN was superior to other super-resolution methods according to the image evaluation indicators (PSNR, SSIM, LPIPS). Moreover, in terms of detail, RFB-ESRGAN had more detailed features and the best performance in the three planes. The results are shown in Figure 11.

### 4.2. MRI Reconstruction Comparison

**Figure 12:**
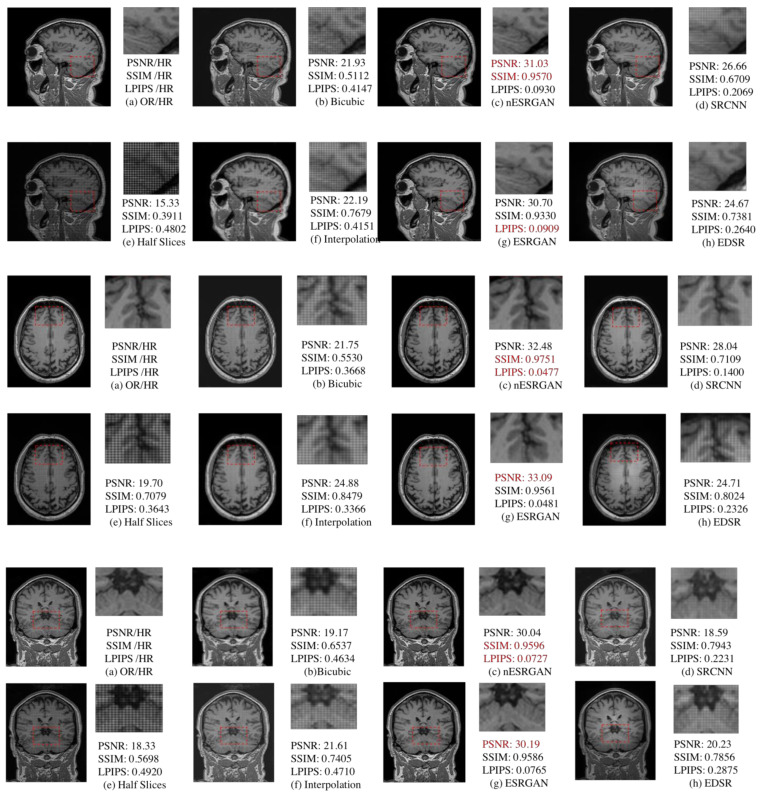
Comparison of bicubic, interpolation [38], advanced methods, and nESRGAN. Among them, (**a**) is the original high-resolution MRI slice, (**e**) is the slice from reconstructed MRI in the first reconstruction. (**b**,**f**) are new slices after interpolation repair. Besides, (**d**,**g**,**h**) are advanced super-resolution methods in deep learning. (**c**) is our proposed method nESRGAN(nosed-based enhanced super-resolution generative adversarial network). (**b**,**d**,**f**,**h**) still exits a few noises and grid lines which missing value lead. (**c**,**g**) perform better than others. The performance of visual quality between (**c**) and (**d**) is similar. Furthermore, (**c**) performs better than (**d**) in the image quality evaluation.

After using RFB-ESRGAN for the first reconstruction, we performed the second MRI reconstruction. The recombined MRI image was very noisy and had missing values. Our previous work involved repairing images according to the principle of linear interpolation, using effective pixel value interpolation instead of null values to obtain a new high-resolution MRI image. Nevertheless, our method still has a small amount of noise, and the visual effect is average [37]. Therefore, we compared our proposed nESRGAN with the previous work. After reconstruction, we tested three slices under three planes. As shown in Figure 12, the performance of nESRGAN was far better than our previous work [38]. At the same time, we also compared nESRGAN with advanced super-resolution methods. The MRI image reconstructed by SRCNN had a different brightness between adjacent slices and insufficient detail information. EDSR recovered some detailed information, but there was still noise. Overall, nESRGAN performs better than other methods in visual quality and image evaluation (Figure 12). Based on nESRGAN, we reconstructed the image after the first rebuilding. We realized super-resolution reconstruction of MRI images on the two-dimensional level through the two networks’ reconstruction work, successfully replacing the three-dimensional convolutional neural network.

### 4.3. Comparison of 2D and 3D

**Figure 13:**
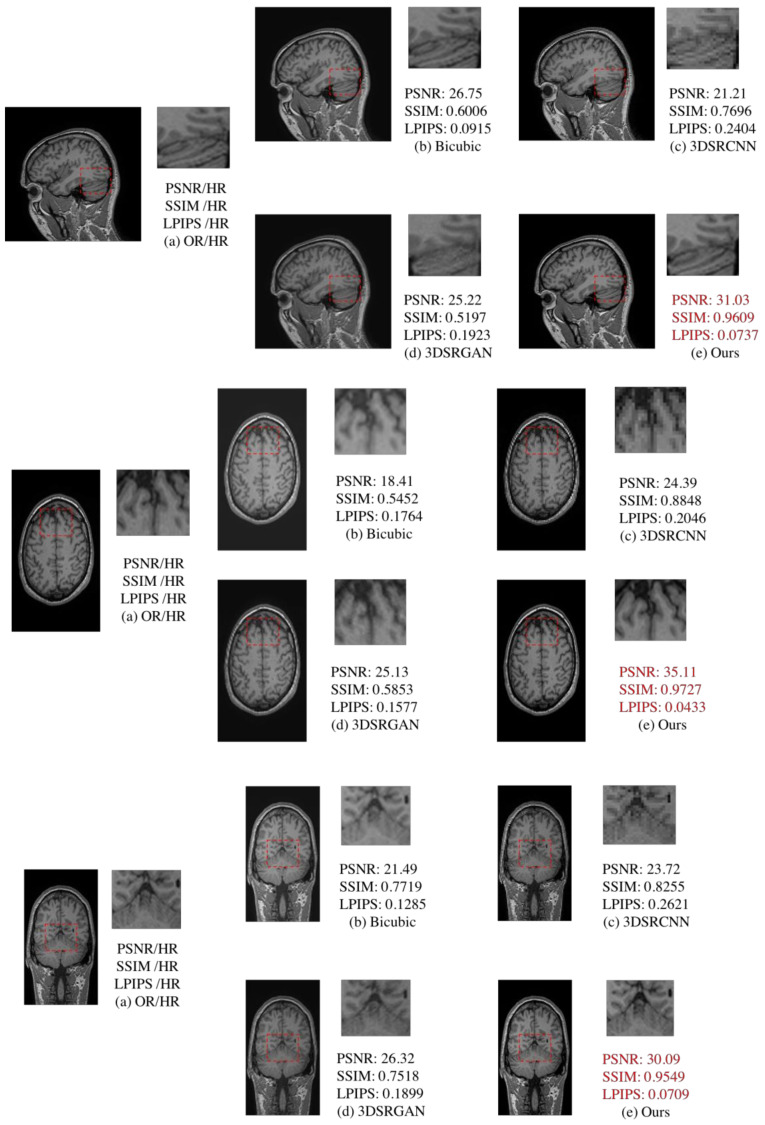
Comparison of 3DSRCNN [21], 3DSRGAN [22], bicubic, and proposed method. (**a**) is the original high-resolution MRI slice. (**b**) is new slice after interpolation of bicubic. (**c**,**d**) are 3D super-resolution methods in deep learning. From this comparison, (**e**) is our proposed method which includes two super resolution reconstruction steps (RFB-ESRGAN and nESRGAN). (**e**) performs better than (**c**,**d**) in the scope of high-frequent information and visual quality. Besides, (**e**) also get better performance in the image quality evaluation.

Three-dimensional reconstruction of MRI images can usually be carried out with a three-dimensional convolutional neural network [22,23,24]. We also compared traditional 3D MRI super-resolution reconstruction methods [22,23] (Table 3). Compared with 3DSRCNN and 3DSRGAN, our approach maintains advantages in image evaluation and detail comparison (Figure 13).

## 5. Discussion

The proposed method employs a two-step 2D super-resolution model to reconstruct 3D MRI images in multiple steps. Figure 2 shows the main idea of restructuring after super-resolution. The method we propose in Figure 4 is to improve the resolution based on computational cost. In the super-resolution method of supervised learning, the paired LR-HR data are prepared for research. Brain MRI images contain all kinds of complex and valuable information. Good pairing of MRI data means that it is necessary to recover as much high-frequency information as possible within limited conditions (time, cost of computing). The RFB-ESRGAN we used (Figure 5) is better than other reconstruction methods in acquiring image detail features (Figure 11). It can be seen from Table 1 that RFB-ESRGAN is basically better than all methods in various image evaluation indicators (PSNR, SSIM, LPIPS). In combination with Figure 7, RFB-ESRGAN also achieves an excellent visual effect without artifacts and local blur. The work of traditional super-resolution reconstruction includes the process of image degradation. Based on this condition, our research adopted slice processing in the initial preparation. Under the condition of 2× mapping combined with the three-dimensional MRI features, we need to treat the input as an image with only half the volume, and the corresponding slices are halved. For this reason, we adopted MRI to separate the pieces. Using RFB-ESRGAN to improve the resolution of half the data (Figure 2), the reconstructed high-resolution slices are reconstituted with MRI (Figure 1), and half of the practical pixel values are missing.

Based on the reconstructed image, our first job is to improve the image resolution while recovering the missing details as much as possible. The last stage in Figure 2 shows the reconstructed image. Traditional interpolation repair generally uses the effective surrounding image pixels to generate the gray value of unknown pixels. However, we tried interpolation in our previous work and the effect was not apparent. As seen in Figure 12, the interpolation method only improves a small part of image. To this end, we need to use super-resolution again for image restoration and resolution enhancement. In the second stage of preparation, we analyzed the role of noise in countering the neural networks. Adding a little noise to the network does not affect the overall amount of calculation and gives the image a little detail. We verified the effectiveness of noise, as shown in Table 2. To avoid the checkerboard effect, we found that interpolation sampling can solve the problem. Our goal is to reduce the computational cost as much as possible and having fewer network parameters can help with this.

For this reason, we also discussed the depth of the two networks. From Figure 4, it can be seen that extending the depth can improve the image efficiency. However, it can be seen from Table 2 that depth enhancement also affects the image reconstruction quality. Simultaneously, we are considering that the depth of the network is minimized to reduce the amount of calculation, so we chose 16 as the depth of the residual in the residual depth network. As shown in Figure 12, the MRI image reconstructed by nESRGAN is indeed superior in visual and image evaluation compared to the other methods. The addition of noise can restore a small amount of detail. Using nESRGAN, the restructured image can be reconstructed with super-resolution again. We use the MRI slice as a degraded image in the data preparation stage and add a downsampling module to the network for feature extraction. The reconstructed MRI slice is directly reconstructed for the second time, and finally the super-resolution reconstruction of 3DMRI is realized.

As shown in Table 3, the method we have proposed in the research is superior to some 3D convolutional neural networks in all aspects. The study shows that it is feasible to reconstruct images twice through super-resolution. Reducing the time cost and computational complexity can help medical staff improve diagnosis and treatment efficiency within a limited time.

## 6. Conclusions

In our experiments, we combined two deep adversarial neural networks to perform the 3D reconstruction of MRI images. We used RFB-ESRGAN based on perceptual information to obtain images with high resolution and a high level of detailed features. After reconstructing the MRI slices, we reorganized the three-latitude high-resolution slices into a three-dimensional MRI image. Then we performed the traversal slice operation again and completed a second reconstruction through the proposed nESRGAN. Finally, we rebuilt the reconstructed new MRI slice for the second time, and finally achieved high-resolution images. Our method is better than traditional 3D super-resolution reconstruction technology in terms of visual effects and image evaluation. In conclusion, the approach we propose successfully achieved 3D reconstruction of MRI images in a 2D field. The reconstructed new images have specific significance in medical diagnosis. Our proposed method only realizes the reconstruction of MRI under the condition of limited zoom factor. In the future, we will try to reconstruct with any size scaling factor, and we also would like to reconstruct MRI based on unsupervised learning.

## Figures and Tables

**Table 1:** Comparison of super-resolution methods on CNN/GAN with 2D MRI images (average ± standard deviation). Our approach achieves best performance in three planes. Red font indicates best performance, blue font indicates second best performance. PSNR, peak signal-to-noise ratio; LPIPS, learned perceptual image patch similarity. FSRCNN [27] is enhanced method based on SRCNN, and EDSR is first method of using deep residual network [15].

		LR	CNN		Deep Network	GAN		
Plane/ Method	Evaluation	Bicubic	SRCNN	FSRCNN	EDSR	SRGAN	ESRGAN	RFB-ESRGAN
Sagittal	PSNR ↑	25.13	14.40 ± 0.22	25.77 ± 0.56	25.62 ± 0.31	26.45 ± 0.64	25.37 ± 0.45	26.20 ± 0.59
	SSIM ↑	0.8106	0.3196 ± 0.0095	0.8337 ± 0.0041	0.8963 ± 0.0035	0.8959 ± 0.0057	0.8856 ± 0.0054	0.9201 ± 0.0041
	LPIPS ↓	0.1996	0.3228 ± 0.0127	0.2140 ± 0.0044	0.1501 ± 0.0043	0.1522 ± 0.0064	0.1525 ± 0.0054	0.1411 ± 0.0043
Coronal	PSNR ↑	26.95	14.10 ± 0.37	29.25 ± 0.64	27.70 ± 0.80	29.16 ± 0.79	29.15 ± 0.86	29.94 ± 0.83
	SSIM ↑	0.7491	0.4175 ± 0.0516	0.7732 ± 0.0124	0.9430 ± 0.0047	0.9372 ± 0.0067	0.9366 ± 0.0049	0.9641 ± 0.0032
	LPIPS ↓	0.149	0.2411± 0.0127	0.1884 ± 0.0095	0.1006 ± 0.0087	0.1077 ± 0.0064	0.0807 ± 0.0092	0.0750 ± 0.0075
Axial	PSNR ↑	29.19	16.46 ± 0.44	28.42 ± 1.12	28.85 ± 1.09	27.69 ± 0.92	27.12 ± 0.88	30.69 ± 0.83
	SSIM ↑	0.8115	0.3682 ± 0.0301	0.8224 ± 0.0452	0.9417 ± 0.0056	0.9027 ± 0.0157	0.9083 ± 0.0089	0.9600 ± 0.0031
	LPIPS ↓	0.131	0.2519 ± 0120	0.2035 ± 0.0174	0.1005 ± 0.0088	0.1148 ± 0.0088	0.1104 ± 0.0079	0.0839 ± 0.0074

**Table 2:** Comparison of configurations in noise based enhanced super-resolution generated adversarial neural networks (nESRGAN). Red font indicates best performance, blue font indicates second best performance (mean ± standard deviation).

		Depth of Residual in Residual Dense Block		Configuration		Original
Plane/Network	Evaluation	23 Blocks Noise/Bilinear	16 Blocks Noise/Bilinear	23 Blocks Noise	23 Blocks Bilinear	23 Blocks
sagittal	PSNR↑ SSIM ↑ LPIPS↓	26.36 ± 1.01 0.9289 ± 0130 0.1255 ± 0.0138	29.63 ± 0.83 0.9478 ± 0.0070 0.0955 ± 0.0124	29.58 ± 1.09 0.9441 ± 0.0052 0.1034 ± 0.0083	29.83 ± 1.08 0.9450 ± 0.0056 0.1063 ± 0.0077	29.28 ± 1.38 0.9452 ± 0.0064 0.1116 ± 0.0080
coronal	PSNR↑ SSIM ↑ LPIPS↓	29.42 ± 1.84 0.9353 ± 0.0033 0.0714 ± 0.0054	32.12 ± 1.23 0.9728 ± 0.0025 0.0586 ± 0.0056	33.36 ± 1.37 0.9510 ± 0.0056 0.0582 ± 0.0047	32.17 ± 1.34 0.9584 ± 0.0018 0.0543 ± 0.0044	33.18 ± 1.45 0.9596 ± 0.0020 0.0533 ± 0.0048
transverse	PSNR↑ SSIM ↑ LPIPS↓	29.16 ± 0.69 0.9222 ± 0.0016 0.0896 ± 0.0055	31.33 ± 0.34 0.9626 ± 0.0018 0.0731 ± 0.0040	31.32 ± 0.46 0.9406 ± 0.0020 0.0799 ± 0.0040	31.04 ± 0.51 0.9435 ± 0.0018 0.0802 ± 0.0039	31.17 ± 0.70 0.9438 ± 0.0024 0.0877 ± 0.0052

**Table 3:** Comparison of 2D and 3D methods (mean ± standard deviation). Red font indicates best performance; blue font indicates second best performance. Our proposed method performs better than 3D methods.

Plane/Network	Evaluation	Bicubic	3DSRCNN	3DSRGAN	Ours
Sagittal	PSNR ↑	25.77 ± 1.32	19.93 ± 0.9728	23.74 ± 1.13	30.28 ± 0.59
	SSIM ↑	0.8170 ± 0.0191	0.7240 ± 0.0346	0.7288 ± 0.0145	0.9497 ± 0.0020
	LPIPS ↓	0.1321 ± 0.0103	0.3288 ± 0.0150	0.2236 ± 0.0102	0.0806 ± 0.0039
Coronal	PSNR ↑	19.44 ± 2.12	24.02 ± 0.72	24.74 ± 1.34	34.25 ± 1.34
	SSIM ↑	0.6318 ± 0.0315	0.8838 ± 0.0183	0.6422 ± 0.0287	0.9710 ± 0.0022
	LPIPS ↓	0.1550 ± 0.0265	0.2300 ± 0.0127	0.1723 ± 0.0149	0.0498 ± 0.0059
Axial	PSNR ↑	23.71 ± 1.69	25.08 ± 1.73	27.43 ± 1.84	30.93 ± 0.90
	SSIM ↑	0.6901 ± 0.0299	0.8634 ± 0.0642	0.7065 ± 0.0549	0.9596 ± 0.0053
	LPIPS ↓	0.1236 ± 0.0233	0.2471 ± 0.0234	0.1486 ± 0.0272	0.0731 ± 0.0121

## Data Availability

Data was obtained from Brain Development and are available https://brain-development.org/ixi-dataset (accessed on 20 August 2020) with the permission of CC BY-SA 3.0 license.
